# Decreased ability in the segregation of dynamically changing vowel-analog streams: a factor in the age-related cocktail-party deficit?

**DOI:** 10.3389/fnins.2014.00144

**Published:** 2014-06-12

**Authors:** Pierre Divenyi

**Affiliations:** ^1^Department of Music, Center for Computer Research in Music and Acoustics, Stanford UniversityStanford, CA, USA; ^2^Speech and Hearing Research, Veterans Affairs Northern California Health Care SystemMartinez, CA, USA

**Keywords:** speech perception, aging, auditory scene analysis, formants, frequency modulation

## Abstract

Pairs of harmonic complexes with different fundamental frequencies f0 (105 and 189 Hz or 105 and 136 Hz) but identical bandwidth (0.25–3 kHz) were band-pass filtered using a filter having an identical center frequency of 1 kHz. The filter's center frequency was modulated using a triangular wave having a 5-Hz modulation frequency f_mod_ to obtain a pair of vowel-analog waveforms with dynamically varying single-formant transitions. The target signal S contained a single modulation cycle starting either at a phase of −π/2 (up-down) or π/2 (down-up), whereas the longer distracter N contained several cycles of the modulating triangular wave starting at a random phase. The level at which the target formant's modulating phase could be correctly identified was adaptively determined for several distracter levels and several extents of frequency swing (10–55%) in a group of experienced normal-hearing young and a group of experienced elderly individuals with hearing loss not exceeding one considered moderate. The most important result was that, for the two f0 differences, all distracter levels, and all frequency swing extents tested, elderly listeners needed about 20 dB larger S/N ratios than the young. Results also indicate that identification thresholds of both the elderly and the young listeners are between 4 and 12 dB higher than similarly determined detection thresholds and that, contrary to detection, identification is not a linear function of distracter level. Since formant transitions represent potent cues for speech intelligibility, the large S/N ratios required by the elderly for correct discrimination of single-formant transition dynamics may at least partially explain the well-documented intelligibility loss of speech in babble noise by the elderly.

## Introduction

In aging various auditory functions of the individual are often impaired. Perhaps the most disturbing aspect of this impairment is the significantly reduced ability to understand speech in social noise or a reverberant environment, commonly referred to as the loss of the “cocktail-party effect” (CPE). Although, expectedly, this deficit is exacerbated by presbycusic—the typical age-related sensorineural high-frequency elevation of auditory thresholds (Carhart and Tillman, 1970)—it is often also experienced by elderly individuals with normal audiograms or having, at worst, a mild-to-moderate hearing loss (Dubno et al., 1984; Divenyi and Haupt, 1997; Snell et al., 2002). Causes of the CPE deficit in the elderly are complex. On the peripheral end, hearing loss has been for long known to affect speech understanding in babble noise, regardless of age (Humes et al., 1994). On the other end of the spectrum, age-related cognitive decline has also been implicated, be it decreased selective attention to concurrent speech (Sommers, 1997), impaired short-term recall of words (Murphy et al., 2000), or reduced working memory capacity (Ng et al., 2013). These factors are among those recognized to increase in the mental effort required when the elderly listens to speech in a CPE setting (Zekveld et al., 2011). But, between peripheral and cognitive extremes there is a host of sensory/nervous system processes indispensable for understanding speech in interference that are also deficient. One group of these are deficits of temporal processing in diverse time ranges, such as gap detection and discrimination necessary for the perception of stop consonants and affricates (Snell and Frisina, 2000), duration discrimination (Fitzgibbons and Gordon-Salant, 1994) affecting accurate perception of subsyllabic and syllabic segments, temporal modulation transfer functions (He et al., 2008) and resistance to modulation interference (Bacon and Takahashi, 1992; Humes et al., 2013), formant transition discrimination (Elliott et al., 1989), and temporal-order discrimination (Fitzgibbons and Gordon-Salant, 1998). A second group is related to localization, which is also known to be impaired in aging (Herman et al., 1977; Abel et al., 2000). This impairment makes CPE performance poorer by reducing or altogether canceling the 2.5-to-4 dB release from masking provided by spatial separation of the target and the interference (Ihlefeld and Shinn-Cunningham, 2008).

From a strictly auditory standpoint, CPE can be regarded as an instance of masking with target speech as the signal and interference as the noise. However, since in speech the respective frequency ranges of the target and the interference seldom perfectly overlap, the rules derived from decades' worth of tone-in-noise *energetic* masking research will be applicable only to specific speech segments. As proposed by authors investigating CPE in laboratories across different continents (Brungart et al., 2001; Cooke et al., 2008) the overwhelming portion of speech in babble noise the masking is *informational*, due to the similarity of the target and the interference (Lee and Richards, 2011). Research over the last few decades uncovered many aspects of informational masking (Watson, 1987; Lutfi, 1990; Kidd et al., 1994; Oh and Lutfi, 1998; Freyman et al., 2004) and was able to quantitatively specify the differences between the two modes of masking (Arbogast et al., 2002; Brungart and Simpson, 2002; Durlach et al., 2003b). As to informational masking in aging, some studies have shown elderly listeners to be more affected than the young (Freyman et al., 2008; Rajan and Cainer, 2008), while others found no age differences (Agus et al., 2009; Ezzatian et al., 2011). The disagreement between these results may stem from the lack of uniformly accepted definition of informational masking other than being different from energetic masking—a tautology pointed out by Durlach (Durlach et al., 2003a)—but also from the inherent difficulty of controlling for the ensemble of physical parameters of speech. However, the lack of age effect could also be due to the elderly listener using his/her experience to compensate for a low speech-to-noise ratio by relying on predictions derived from overlearned patterns (Divenyi, 2005).

CPE can be also viewed as the instance of auditory scene analysis (ASA, Bregman, 1990) most important for verbal communication. In fact, in a CPE situation the listener must continuously segregate a speech target stream from the babble stream or streams. While young normal-hearing individuals are able to understand speech in CPE settings even under quite unfavorable signal-to-noise ratios (SNR's), the SNR elderly individuals require is significantly higher (Gelfand et al., 1988; Snell et al., 2002), even when these individuals suffer from no or only mild presbycusic hearing loss (Divenyi and Haupt, 1997). The way ASA understands speech segregation of target from non-target speech is that harmonics of the fundamental frequency (f0) each of the simultaneous voices are grouped, thereby allowing the listener to focus on the harmonics of the target voice alone—as demonstrated by experiments on the segregation of non-speech harmonic complexes (Micheyl and Oxenham, 2010) and synthesized as well as natural speech sounds (Darwin et al., 2003; Roman and Wang, 2006; Lavandier and Culling, 2008). Segregation of concurrent vowels (Assmann and Summerfield, 1990), or speech of concurrent talkers (Darwin et al., 2003), is easier when their f0's are widely separated (e.g., as in the voices of different gender talkers) and becomes increasingly difficult as the difference between f0's decreases. Temporal asynchrony of vowels (Darwin and Hukin, 1998) or words (Lee and Humes, 2012) also facilitates their segregation. The ability to segregate one vowel in an ensemble of concurrent vowels increases when the f0 of one or several in the ensemble is modulated by a low-frequency sinusoid (i.e., when it undergoes a *vibrato*) (McAdams, 1990). In rooms, the target and non-target talkers are in spatially separated locations allowing the auditory system to segregate them, as shown in binaural experiments (Brungart and Simpson, 2002, 2007; Hawley et al., 2004).

But, looking from a broad perspective, speech is a dynamic signal characterized by constant *changes*. The changes can be defined in various ways, such as on the level of acoustics (fluctuating envelope, fundamental frequency variations, formant transitions, etc.), articulatory phonetics (gestural movements), descriptive phonetics and phonology (sequences and clusters of phonemic and sub-phonemic units), or higher-order linguistics (sequences of morphemes, words, word strings, sentences, sentence strings). Although computational characterization, and modeling, of these changes is nearly impossible at the higher levels of analysis, a mathematical formulation of the transform of acoustic signals to activity patterns observed at the cortical level, the complex modulation spectrum based on Gabor's wavelet transform (Gabor, 1946) has been gaining acceptance. Although Gabor conceived it for the reduction of information “atoms” in audio (i.e., telephone) communication, the transform and its inverse have been widely used for the analysis and synthesis of images (Levi and Stark, 1983) before being adopted for the analysis of audio signals (Pitton et al., 1996) and to models of the auditory system beyond peripheral analysis (Kowalski et al., 1996). Recognizing that both the temporal and spectral envelopes of natural (i.e., complex) sounds contain peaks that change over time, the transform represents the *spectrum* of these peaks as *modulations* in the temporal (rate, in Hz) and spectral (scale in cycles per octave) domains. By choosing appropriate parameters for this model, called the “spectro-temporal receptive field” (STRF) model, it has been demonstrated that auditory cortex activity in the ferret (Chi et al., 2005) or in the song bird (Singh and Theunissen, 2003), as well as temporal-parietal cortical responses recorded with an electrode grid placed on the surface of patients awaiting epilepsy surgery (Mesgarani et al., 2008), can be fairly accurately modeled using this transform. In agreement with Plomp (1983)—“… speech is a signal slowly varying in amplitude and frequency”—and with the 4-Hz major mode of the temporal modulation spectrum of speech (Greenberg et al., 2003), shown to be language-independent (see e.g., Arai and Greenberg, 1997) speech input to this model shows that the predominant temporal modulation rate is slow and so is the scale of frequency peak shifts during relatively stable segments (e.g., vowels, fricatives, nasals, Elliott and Theunissen, 2009). Because the transform effectively uncovers patterns and features of complex signals—speech, music, animal sounds, and environmental sounds—it has been used as a tool for the separation of concurrent auditory streams in a cocktail-party situation (Elhilali and Shamma, 2008; Mesgarani et al., 2011). Continuing this line of thought, if the auditory system uses temporal and spectral modulations to picture our acoustic world and to separate auditory objects, then studying the perception of signals modulated in amplitude and/or frequency, as well as its impairments, should bring us closer to the understanding of the success and failures of listening in a CPE setting. Thus, data on modulation detection/discrimination interference (MDI) in the amplitude (Moore et al., 1995; Moore and Sek, 1996), or frequency (Lyzenga and Carlyon, 1999) domains not only reveal parametric limitations of the Gabor transform applied to audition but also quantitatively describe the dynamic temporal and spectral map inside the existence limit of the CPE.

The present study continues the above line of reasoning in a set of experiments aimed at better understanding components of the deficiency elderly individuals display when listening to speech in the presence of speech interference. Since an earlier study showed the effect of duration and velocity on the perception of vowel transitions (Divenyi, 2009), and since the perception of frequency transitions in aging has been shown to correlate with intelligibility (Gordon-Salant et al., 2007), the experiments were focused on the way, and the extent to which, identification of a transition of interest is affected by the presence of an similar transition. The experiments used a single-formant simplified analogs of a target vowel and of an interfering vowel, each having a fixed f0 and a formant peak modulated in frequency. A similar stimulus configuration was used in studies by Lyzenga and Carlyon (1999, 2005) focused on the effects of the difference between either modulating or fundamental frequency, and of spectral content. In contrast, the question the present experiments addressed was the target-to-distracter ratio (TDR) necessary for a formant peak modulation pattern to be identified by normal-hearing young, and by elderly listeners without appreciable hearing loss.

## Materials and methods

**Figure 1:**
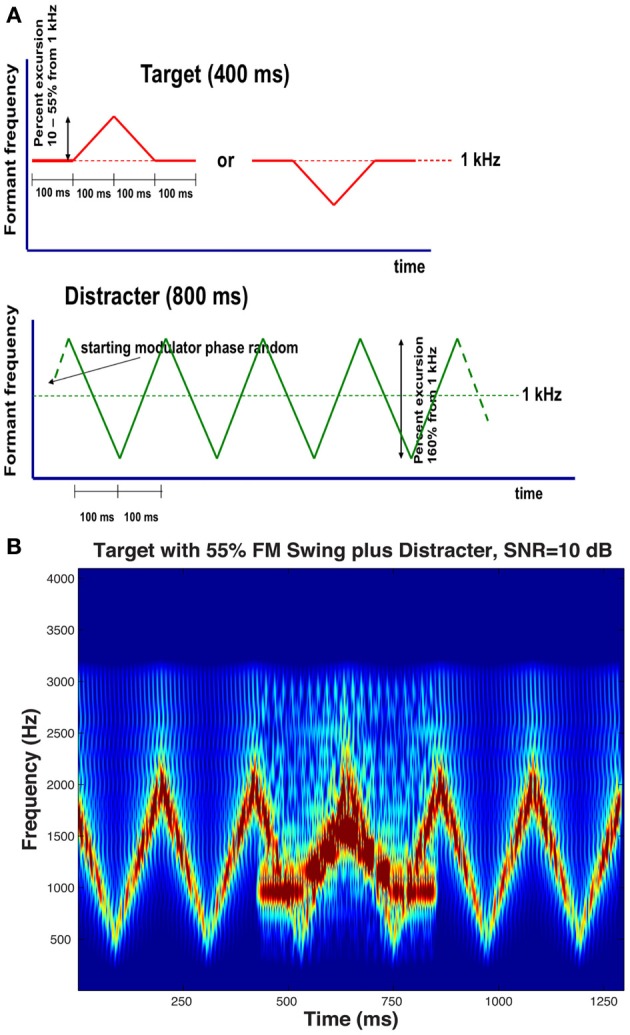
**(A)** Schematic stimulus diagram, illustrating trajectory of the single formant's peak in the target and in the distracter, frequency-modulated using a 5-Hz triangular wave. The upper traces illustrate the two target formant peak trajectory patterns that the listeners had to identify: a 100-ms ascending followed by a 100-ms descending trajectory or a 100-ms descending followed by a 100-ms ascending trajectory, both trajectories flanked by two 100-ms steady-state portions with a constant formant peak at 1 kHz. The lower trace illustrates the trajectory of the distracter that consisted of an 800-ms pattern of repeating 100-ms ascending and descending trajectories between the 525 and 1800-Hz formant peak minimum and maximum that are equidistant from the 1-kHz center on a basilar membrane distance scale. The starting phase of the distracter's modulating waveform varied randomly from trial to trial. All formant trajectories were linear on this distance scale. Both the target and the distracter were gated using a 25-ms cosinusoidal window to ensure that the onset and the offset of both were smooth. **(B)** Spectrogram of a trial. The target is an up-down transition with an FM excursion reaching 1550 Hz, i.e., 55% higher than the resting 1-kHz formant frequency. The SNR of the example shown, 10 dB, is larger than most conditions used in the experiments.

Stimuli in the experiments consisted of pairs of harmonic complexes: a target stream presented simultaneously with an interfering distracter stream. The 800-ms distracter stream started 200 ms before the 400-ms target stream. The two streams had different fundamental frequencies (f0), one always 107 Hz for one of the streams and either 136 or 189 Hz for the other, thereby producing two different fundamental frequency separations (Δf0), one wide (Δf0/f0 = 0.77, approximately corresponding to the minor seventh musical interval) and one narrow (Δf0/f0 = 0.27, approximately corresponding to the major third). At each Δf0 separation, the higher f0 was assigned to the target in half of the conditions while it was assigned to the distracter in the other half. The spectrum of both streams contained only harmonics inside the 250- to 3000-Hz band. Because this constraint resulted in a certain degree of difference between the perceived salience of the two streams, Terhardt's algorithm (Terhardt et al., 1982) was used to generate streams the salience of the dominant temporal (“virtual”) pitch of which was comparable. Both streams were spectrally shaped to produce single-formant pseudo-vowels by passing them through second-order band-pass filters with a 6 dB/octave falloff, i.e., filters with formant characteristics not unlike that of natural vowels. The target stream's formant frequency F_T_ was held constant at 1 kHz during the first and last 100 ms of its duration, while during the central 200 ms the F_T_ was modulated with a 5-Hz triangular wave that went for 100 ms in one direction and for 100 ms in the other, thereby creating formant trajectory patterns in which F_T_ was changing either up-down or down-up starting from, and returning to, an F_T_ of 1 kHz, with a maximum formant swing of ΔF_T_ Hz. In the distracter stream, the formant frequency F_D_ was also modulated with a 5-Hz triangular wave, except that the modulator waveform was during the whole duration of the distracter, creating a continuous up-down-up-down pattern. The extent of the distracter's formant trajectory was also larger than that of the target: the top and the bottom formant frequency extremes were 1800 and 525 Hz, i.e. two frequencies equally distant from 1-kHz, a frequency at the center of the low and high extremes on Greenwood's (1962) scale of basilar membrane distances. Schematic formant frequency-vs.-time diagrams of the target and the distracter are shown in Figure 1A, with an audio example presenting the target, the distracter, and their combination. Throughout the experiments, the starting modulation phase of the distracter randomly varied from trial to trial. Figure 1B displays a spectrogram representing a trial having an up-down target a large, 55%, formant excursion embedded in the distracter; the TDR is +10 dB—a ratio larger than most used in the experiments proper and is shown here mainly for illustrative purposes.

The study included two experiments. The objective of the first was to examine how the ensemble of stimulus parameters affected the threshold of *discriminability* of formant transition patterns. The objective of the second experiment was to examine the effect of the stimulus parameters on the threshold of *detectability* (i.e., audibility) of the target. In Experiment 1, the subject performed a single-interval two-alternative forced choice task that consisted of *identifying* whether the formant trajectory pattern in the target stream was up-down or down-up, while ignoring the distracter stream. In each block of trials ΔF_T_/F_T_, the frequency excursion (i.e., the swing) of the target, remained fixed at 10, 20, 30, or 55 percent and the overall level of the distracter was held constant at 60, 70, or 80 dB SPL. The difference between the fundamental frequencies of the target and the distracter, Δf0/f0, was narrow or wide and varied from condition to condition, and so did the assignment of the higher or the lower of the fundamental frequencies to the target stream (and the other fundamental frequency to the distracter). In Experiment 2 the subject performed a two-interval two-alternative task in which he/she had to *detect* whether the target formant pattern was present in the first or the second interval, with the distracter being presented in both intervals. Two formant swing extents, 10 and 55 percent, were investigated with the distracter level constant at 60, 70, or 80 dB SPL. The higher fundamental frequency was always assigned to the target and the fundamental frequency separation was always the wide one (Δf0/f0 = 0.77).

Stimuli were digitally stored and delivered by a PC computer using an Echo Gina analog converter system connected to Tucker and Davis Technology filters and digital attenuators, and delivered diotically to headphones (Sennheiser SH 250). In each run of trials in both experiments the level of the target stream was varied adaptively from a starting point of 90 dB SPL to track the 79.4% correct performance threshold. The initial step size was 5 dB and was reduced with whenever the subject gave three consecutive correct responses first to 2, and then to 1 dB. The run was terminated at the tenth reversal and threshold in each run was calculated as the average of the target's dB level at the last eight reversals. The threshold estimate for each subject and each condition was the arithmetic mean of thresholds obtained in six to eight runs.

Listener performance was assessed for subjects in two groups. The young group included 17 normal-hearing individuals between 19 and 29 years of age (average 22.0 ± 3.4 years). The elderly group included 12 elderly individuals between 61 and 82 years of age (average 69.0 ± 6.7 years) in Experiment 1, 10 of whom also participated in Experiment 2. Their hearing impairment, when present, was a mild-to-moderate presbycusic sensorineural loss; the mean of the group's pure-tone average thresholds between 0.5 and 4 kHz was 19.3 ± 14.2 dB SPL. Several elderly subjects had normal hearing and none had hearing loss exceeding 25 dB under 3 kHz, that is, in the frequency region of the stimuli. Subjects were tested individually in sessions that lasted 1 h a day. All subjects received training with stimuli used in both experiments. Data collection for each subject was started after he/she obtained a score of at least 95 percent correct in two contiguous 60-trial runs using the largest formant excursion (55%) without the distracter present, and a score of at least 80 percent correct in two 60-trial runs with the distracter present at 60 dB SPL, using the 55% formant excursion, and a constant target level of 80 dB SPL. Typically, young subjects needed one-and-half session to reach these criteria, whereas elderly subjects needed, on the average, two-and-half sessions. Subject testing procedures were fully consistent with experimental protocols approved by the V.A. Northern California Health Care System's Institutional Review Board.

## Results

### Experiment 1—identification of formant trajectory patterns

**Figure 2:**
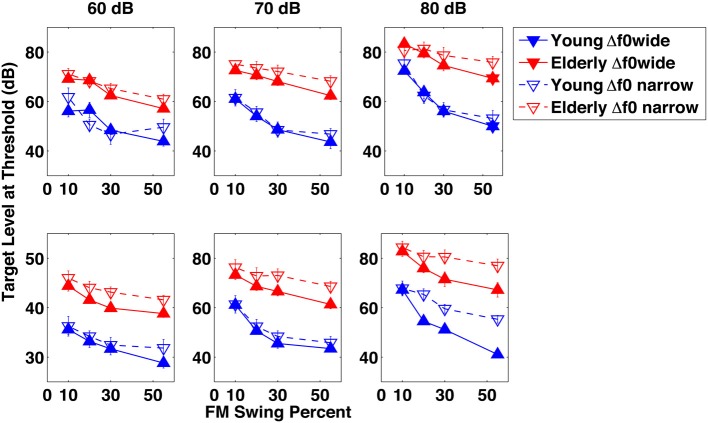
**Results of Experiment 1**. Target level at identification threshold in dB as a function of the target formant's frequency modulation swing expressed as percent maximum displacement from 1 kHz, across three distracter levels in dB, in separate columns of graphs. The elderly group's data are in red and those of the young group in blue. Filled symbols represent results for conditions in which the fundamental frequency separation Δf0/f0 between the target and the distracter was wide (0.77), whereas data marked by the empty symbols are for conditions with a narrow (0.27) Δf0/f0. In the top row of graphs the data shown represent for conditions in which the fundamental frequency f0 of the target was always *lower* than that of the distracter, whereas in the bottom row of graphs they represent conditions in which the fundamental frequency f0 of the target was always *higher* than that of the distracter.

Figure 2 illustrates the results of the experiments on the identification of a dynamically changing single- and formant target pattern in the background of a dynamically changing single-formant distracter. The figure represents threshold level of the target pattern for the average of the elderly (red lines and symbols) and the young (blue lines and symbols) subject groups, as a function of the extent of the target formant's excursion across the three distracter levels. All panels compare results of the wide (Δf0/f0 = 0.77, approximately minor seventh, solid lines) and narrow (Δf0/f0 = 0.27, approximately major third, dashed lines) f0 separations. In the top panels the target's f0 is lower and in the bottom panels it is higher than that of the distracter.

Looking at the young group's data within each and across all of the four figure panels, several general observations can be made (lower target thresholds indicating better performance):

1. Increasing distracter level from 60 to 70 and 80 dB resulted in increased target levels. For a 20 dB increase in the distracter level a 10.6 dB target level increase was required, suggesting that the target level was a compressed nonlinear function of the distracter level.
2. Decreasing the excursion resulted in an increase of the target threshold, although substantial increase was seen mainly at the smallest (10%) excursion extent. The difference between target levels at the easiest (55%) and hardest (10%) swing extents averaged across all conditions was large (16.2 dB). Because most of the target level increase occurred between the two smallest swings (10% and 20%), the target level was an expansive nonlinear function of the formant excursion.
3. At both f0 separations, when the target's f0 was lower than distracter's the task was easier, resulting in target thresholds 2.75 dB lower on the average across all conditions.
4. The large f0 separation was easier than the narrow one, resulting in target thresholds 4.6 dB lower on the average across all conditions.
5. At the threshold of identifiability, the TDR was −22.40 dB at the easiest and −4.61 dB at the hardest condition, that is, the level of the target was below that of the distracter even when identifiability of the target was most difficult.

The trend of the elderly subjects' data mirrors that of the young subjects. In general, the differences within the elderly group's data with regard to swing, f0 separation, and target f0 are comparable to, or somewhat smaller than, those exhibited by the young subject group. The target level-distracter level nonlinearity for the elderly is a little smaller than for the young: a 12.6 dB target level increase for a 20 dB distracter level increase. Averaged across conditions, the target level difference between the easiest and hardest swing conditions was 9.6 dB, between the assignments of the higher f0 to the target or to the distracter was 4.02 dB, and between the two f0 separation was a mere 0.2 dB. However, when comparing the elderly and the young groups' results, one striking feature appears: the elderly subjects' target levels are about 20 dB higher than those of the young subjects, in all experimental conditions. In other words, in order to identify the target formant pattern as up-down or down-up, elderly listeners needed a target intensity about 20 dB higher than the young, regardless of the condition. Expressing the results as TDR, average TDR for the young was −22.4 dB in the easiest and −4.60 in the most difficult condition, whereas TDR for the elderly was −2.85 and 4.34 dB, respectively, for the two difficulty degrees. [The easiest condition was that of the 60 dB SPL distracter, the widest (55%) swing, the wide f0 separation, and for the target having the higher f0 than the distracter. In the same vein, the hardest condition was that of the 80 dB SPL distracter, the narrowest (10%) swing, the narrow f0 separation, and for the target having the lower f0 than the distracter]. Thus, the elderly-young discrepancy when the task is easy is the same 20 dB as for the overall data shown in the figures but it diminishes to only 8.3 dB when the tasks are difficult.

To uncover details and to analyze the statistics of the observations, an analysis of variance was conducted. Results of the ANOVA are shown in Table 1. As the probability (p-) column indicates, all main effects—distracter level, formant swing extent, the size of f0 separation, and assignment of the higher f0 to target or distracter—were highly significant with *p* = 0.0001, both within and across subjects. The subject group effect was also highly significant and so were the within subject and main effect interactions, except the within subject-f0 separation effect, indicating that some subjects found the task for both Δf0/f0's equally difficult or easy. The significant interaction between both individual subjects and subject group vs. the assignment of the higher f0 to target or distracter indicates, as suggested by Figure 2, that the elderly, as well as some individual subjects, found it more consistently easier to identify the target pattern when the target f0 was the higher one. The significant interaction between formant swing extent and f0 assignment indicates, as both rows of graphs in Figure 2 illustrate, that the target's f0 assignment to the higher f0 made the task easier only when the swing was relatively large, i.e., when the task itself was less difficult. The lack of significance of the Δf0/f0-distracter level and the Δf0/f0-subject group interactions indicate that frequency separation was a stable effect unaffected by the loudness of the distracter or the age of the listener.

**Table 1:** **ANOVA of target identification data**.

**Analysis of variance**					
**Source**	**Sum sq**.	***df***	**Mean sq**.	***F***	**Prob > *F***
SUB(Sgroup)	58931.9	27	2182.7	77.92	0
Dlevel	28,678	2	14,339	511.91	0
Trghi/Lo	1941.7	1	1941.7	69.32	0
Df0	622.8	1	622.8	22.23	0
Swing	33,475	3	11158.3	398.36	0
Sgroup	48032.7	1	48032.7	1714.79	0
SUB(Sgroup) * Dlevel	6475.1	54	119.9	4.28	0
SUB(Sgroup) * Trghi/Lo	2826.3	27	104.7	3.74	0
SUB(Sgroup) * Df0	737.6	27	27.3	0.98	0.5011
SUB(Sgroup) * Swing	6641.1	81	82	2.93	0
Dlevel * Trghi/Lo	485.4	2	242.7	8.66	0.0002
Dlevel * Df0	13.4	2	6.7	0.24	0.7869
Dlevel * Swing	685.6	6	114.3	4.08	0.0005
Dlevel * Sgroup	1161.3	2	580.7	20.73	0
Trghi/Lo * Df0	194.4	1	194.4	6.94	0.0085
Trghi/Lo * Swing	1982.1	3	660.7	23.59	0
Trghi/Lo * Sgroup	1292.2	1	1292.2	46.13	0
Df0 * Swing	386.9	3	129	4.6	0.0033
Df0 * Sgroup	89.6	1	89.6	3.2	0.0739
Swing * Sgroup	956.2	3	318.7	11.38	0
Error	32016.3	1143	28		
Total	229346.1	1391			

*Factors: Sgroup—elderly/young groups; Dlevel—Distracter level; Targhi/Lo—f0 of target higher or lower than f0 of distracter; Df0—f0 separation wide/narrow; Swing—Maximum of target formant's excursion*.

### Experiment 2—comparison target pattern detection and identification

**Figure 3:**
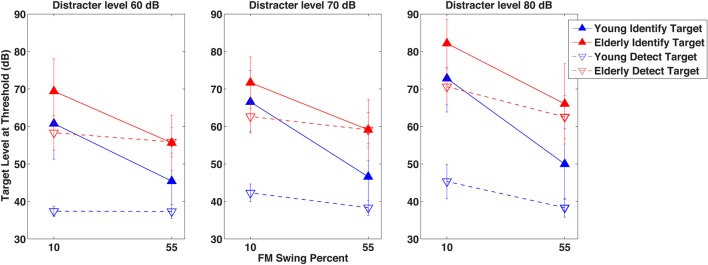
**Results of Experiment 2**. A comparison of detection and identification of the target in the presence of the distracter. As in Figure 2, target level at threshold is shown in dB as a function of the target formant's FM excursion expressed as percent maximum displacement from 1 kHz. The three separate graphs indicate data for three distracter levels in dB. Unfilled symbols represent threshold levels for the *detection* of the target, whereas filled symbols represent thresholds for the *identification* of the target. The fundamental frequency separation Δf0/f0 of the target and the distracter was *wide* (0.77) and the fundamental frequency f0 of the target was always *higher* than that of the distracter.

After seeing the subjects' performance in identifying the correct formant pattern, one is compelled to ask the question just how detectible the patterns were. This question was put to test in Experiment 2 in which detectability of a small number of selected target patterns was measured when its audibility was masked by the distracter used in Experiment 1. Figure 3 illustrates with the closed symbols data of the detection experiment. The same subjects' results in the identification experiment at the same conditions are also shown for comparison with the open symbols. Elderly subjects needed the target level to be 18.6 dB higher than the young at the 60 dB SPL distracter level and 24.22 dB higher at the 80 dB SPL distracter level. At the easy (55% swing) conditions detection of the target for the young subjects required a substantially lower (between 8.1 and 11.6 dB) target level than did its identification, whereas for the elderly subjects the two tasks required the same level, except at the most intense distracter level, where identification level was 3.5 dB higher than the detection level. Contrary to identifiability of the target (as seen in Experiment 1), the extent of formant swing did not change its detectability: the swing had no influence on whether the target could be heard. Analysis of variance of these results, shown in Table 2, uncovered highly significant main effects and, except for distracter level, also within subject-main effect interactions. The highly significant subject group- task (detection vs. identification) and individual subject-task interactions indicate that a definite age effect for the way detection and identification are performed (and perhaps also understood). The significant group-formant swing and group-distracter level interactions show that elderly and young listeners are differentially affected by the difficulty of the task, be it detection or identification. The highly significant task-by-swing and task-by-distracter level interactions mean that factors making identification easier or more difficult had no bearing on detection, i.e., once a target was audible, many of its properties were irrelevant. This conclusion seems to have been shared by the two groups, as indicated by the non-significant subject group-by-task interaction.

**Table 2:** **ANOVA of data from detection vs. identification tasks**.

**Analysis of variance**					
**Source**	**Sum sq**.	***df***	**Mean sq**.	***F***	**Prob > *F***
SUB(Sgroup)	8922.3	25	356.9	11.86	0
Sgroup	8352.9	1	8352.9	277.67	0
Dlevel	285.8	2	142.9	4.75	0.0097
Det/Ident	14427.4	1	14427.4	479.61	0
Swing	2724.9	1	2724.9	90.58	0
SUB(Sgroup) * Dlevel	1855.4	50	37.1	1.23	0.1612
SUB(Sgroup) * Det/Ident	3781.2	25	151.2	5.03	0
SUB(Sgroup) * Swing	605.9	25	24.2	0.81	0.7318
Sgroup * Dlevel	223.2	2	111.6	3.71	0.0263
Sgroup * Det/Ident	19.3	1	19.3	0.64	0.4242
Sgroup * Swing	303	1	303	10.07	0.0018
Dlevel * Det/Ident	3686.5	2	1843.3	61.28	0
Dlevel * Swing	247.3	2	123.7	4.11	0.0179
Det/Ident * Swing	9287.4	1	9287.4	308.74	0
Error	5535.1	184	30.1		
Total	60526.2	323			

*Factors: Sgroup—elderly/young groups; Dlevel—Distracter level; Det/Ident—Task (detection vs. identification); Swing—Maximum of target formant's excursion*.

## Discussion

Formant transitions in vowels convey important information. Although a large excursion of transition indicates phonemic change and mostly signals the presence of a diphthong, even a relatively minor dynamic change in the frequency of a formant peak contributes to intelligibility because it is one of the markers of the consonant preceding and following the vowel (Hillenbrand et al., 2001; Fogerty and Kewley-Port, 2009). The present experiments investigated the identifiability and detectability of a simplified form of these transitions in the presence of intense distracter transitions, both in the range of those indicating phonemic change—like the 55% excursions—and those signaling the identity of preceding/following consonants—like the 10–20% excursions. The FM rate chosen, 5 Hz, was also similar to syllabic rate and thus makes the results comparable to speech and to the CPE. From a psychoacoustic standpoint, the results complement the vast and detailed body of information on modulation interference and modulation masking that has established parametric limits for detection and discrimination thresholds of a target in the presence of similar interference, with respect to differences in frequency, modulation rate, level, and some other properties. Although adding to this body was not the primary objective of the present study, it showed that increasing the level of interference resulted in a compressed growth in the level of the target, not only in the identification but also in the detection task. The present experiments used vowel-like harmonic target and distracter—a situation treated by only a relatively small number of studies (e.g., Shackleton and Carlyon, 1994; Lyzenga and Carlyon, 1999, 2005) that examined frequency modulation masking (FMD) for signals with f0's typically closer to each other than even those of our narrow f0 separation. Present in Lyzenga's and Carlyon's data, although not specifically pointed out in their papers, was the finding that FMD was larger when the target f0 was below that of the interferer. The present data shown in Figure 2 clearly show a worse overall performance, especially in the difficult 10% swing conditions, when the target f0 was lower than the distracter's. One explanation could be that when the distracter f0 is higher, it will have more intense harmonics in the frequency range where the target's second-to fifth harmonics (those that are most important for carrying pitch information) are located. Western composers from the Renaissance period on (i.e., from the beginnings of accompanied melody and polyphony) have been well aware of this relationship and have customarily placed the melody intended to be heard in the treble.

Clearly, the important finding of the experiments is the impaired ability by the elderly listeners to detect and identify formant excursions in the target embedded in a distracter. Since deficits have been documented for a variety temporal processing tasks in elderly individuals with little or no presbycusic impairment (Gordon-Salant and Fitzgibbons, 1999; Humes et al., 2012), it is unlikely that our elderly listeners' deficiency may be due to the presence of their not more severe than mild-to-moderate hearing loss. The surprising finding is the large difference, 20 dB on the whole, between target identification thresholds of young and elderly subjects. Thus, one could hypothetically assume that, in addition to a small part attributable to high-frequency threshold elevation that would have diminished the contribution of higher harmonics to the strength of the definition of formants, decline of a more central, possibly cortical, site may account for the observed perceptual loss.

### Relevance of the results

Formant transitions in vowels convey important information. Although a large excursion of transition indicates phonemic change and mostly signals the presence of a diphthong, even a relatively minor dynamic change in the frequency of a formant peak contributes to intelligibility because it is one of the markers of the consonant preceding and following the vowel (Hillenbrand et al., 2001; Fogerty and Kewley-Port, 2009). The present experiments investigated the identifiability and detectability of a simplified form of these transitions in the presence of intense distracter transitions, both in the range of those indicating phonemic change—like the 55% excursions—and those signaling the identity of preceding/following consonants—like the 10–20% excursions. The FM rate chosen, 5 Hz, was also similar to syllabic rate and thus makes the results comparable to speech and to the CPE. From a psychoacoustic standpoint, the results complement the vast and detailed body of information on modulation interference and modulation masking that has established parametric limits for detection and discrimination thresholds of a target in the presence of similar interference, with respect to differences in frequency, modulation rate, level, and some other properties. Although adding to this body was not the primary objective of the present study, it showed that increasing the level of interference resulted in a compressed growth in the level of the target, not only in the identification but also in the detection task. The present experiments used vowel-like harmonic target and distracter—a situation treated by only a relatively small number of studies (e.g., Shackleton and Carlyon, 1994; Lyzenga and Carlyon, 1999, 2005) that examined FMD for signals with f0's typically closer to each other than even those of our narrow f0 separation. Present in Lyzenga's and Carlyon's data, although not specifically pointed out in their papers, was the finding that FMD was larger when the target f0 was below that of the interferer. The present data shown in Figure 2 clearly show a worse overall performance, especially in the difficult 10% swing conditions, when the target f0 was lower than the distracter's. One explanation could be that when the distracter f0 is higher, it will have more intense harmonics in the frequency range where the target's second-to fifth harmonics (those that are most important for carrying pitch information) are located. Western composers from the Renaissance period on (i.e., from the beginnings of accompanied melody and polyphony) have been well aware of this relationship and have customarily placed the melody intended to be heard in the treble.

A comparison of the detection and identification results seen in Figure 3 would be interpreted by some authors (e.g., Brungart et al., 2001) as a contrast between energetic masking and informational masking—energetic masking being considered as the process underlying detection and informational masking as a process of interference not attributable to energetic masking (Durlach et al., 2003a). One particular result, however, is incompatible with the energetic-informational masking contrast: while young listeners needed a higher SNR for identification than for detection regardless of the difficulty of the task, for the easiest condition (when the target has the highest extent of FM swing) the older listeners needed the same SNR for detection and identification.

Clearly, the major finding of the experiments is that the ability by the elderly listeners to identify and also to detect formant excursions in the target embedded in a distracter is impaired. This finding adds to a long list of deficits for a variety temporal processing tasks in elderly individuals who, just as our elderly listeners, had little or no presbycusic impairment (e.g., Gordon-Salant and Fitzgibbons, 1999; Humes et al., 2012). While peripheral auditory impairment could have had some contribution to the 20 dB effect in our results even if the threshold shifts indicated by the elderly person's audiogram were relatively minor, it is likely that some dysfunction higher up on the auditory pathway was more accountable for the loss illustrated in Figure 2. Obviously, it would be of interest to answer the question regarding what proportion of the loss observed in the data is attributable to peripheral and what to central impairment. This question could be addressed empirically by conducting a series of tests on the same subjects to measure a wide range of spectral and temporal auditory capabilities that, according to the literature, could differentiate peripheral and central auditory processing—such as amplitude and frequency modulation transfer functions, auditory filter width, pitch discrimination and salience, temporal processing in the 100-ms and longer ranges, auditory attention, and short-term memory for auditory stimulus details, only to cite a few. Unfortunately, such multidimensional data are not available for the subjects tested in the present experiments and the question can't be answered by analyzing the present data. Borrowing from physics, questions such as ours may be addressed indirectly by computational experiments using simulation. In our case, we could simulate normal and impaired processing of the present stimuli by using a model of the auditory system that includes both peripheral and central stages. The following subsection describes such a simulation with the help of tmodel mentioned in the Introduction, the STRF model (Chi et al., 2005). This model was chosen because it includes a peripheral and a central auditory stage, and because it permits manipulation of the efficacy of both stages.

### Simulation of the results using the STRF model

**Figure 4:**
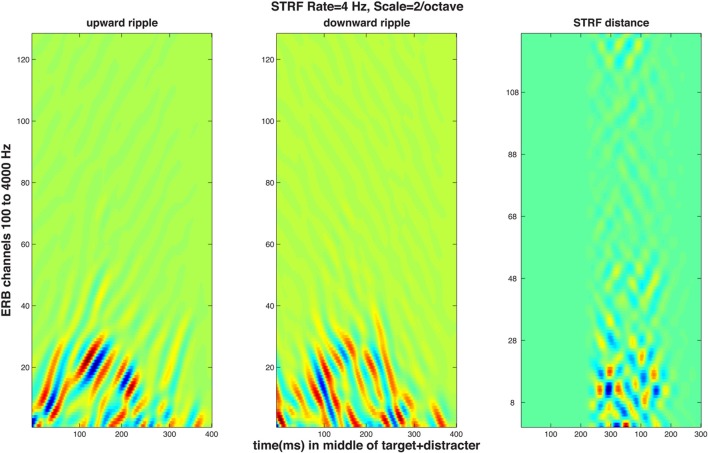
**STRF magnitude responses to a target-plus-distracter stimulus**. The graphs represent simulated cortical response patterns of 128 channels tuned to frequencies aligned on an equivalent rectangular bandwidth (ERB) scale, as a function of time over the 600-ms middle portion of the stimulus containing 600-ms of the distracter and 400 ms of the target (see Figure 1) starting 100 ms after the portion of the distracter shown. All panels refer to STRF activity of regions tuned to 4-Hz temporal modulation rate and 2 cycles/octave spectral modulation. Left and middle panels: upward (left) and downward (middle) time-frequency modulation ripple STRF responses to an up-down target pattern embedded in the distracter, i.e., activity elicited by +4 Hz and −4 Hz temporal modulation, respectively. The rightmost panel displays pixel-by-pixel Euclidean distances of STRF magnitude evoked by the up-down and the down-up target in an identical distracter, i.e., two stimuli differing only in the very center of their duration.

The STRF model first performs a multichannel filtering and compression akin those that take place in the cochlea and the auditory nerve, resulting in an “auditory spectrogram” with a critical band-type ERB (equivalent rectangular band) frequency scale (Patterson and Moore, 1986). This time-frequency response matrix is led to a subsequent stage in which temporal and spectral modulations are analyzed and decomposed to obtain a four-dimensional representation (time, frequency, temporal modulation by the rate of change, and spectral modulation by the scale of adjacent peaks in the spectrum). Such decomposition is known to take place in the cortex of animals (Kowalski et al., 1996) and humans (Mesgarani et al., 2008). The model is well suited for simulation of normal and impaired auditory processing on both levels because changing the threshold and the filtering in the first stage can mimic, to some extent, high-frequency presbycusic loss by the elderly. In the second stage, resolution of temporal and/or spectral modulation (i.e., grating) can be reduced by changing the appropriate parameters. Because of the magnitude of the effect obtained in the results, simulation of only the identification data was performed. It was assumed that identification of the up-down or the down-up pattern was based on the subject computing a distance between the four-dimensional STRF activity evoked by the two targets presented in the distracter. Since only one of the patterns was actually heard, it was further assumed that the STRF of the other target pattern was preserved and kept intact in memory, and it was available for the subject to perform the distance computation between the just-heard “pattern 1” and the previously hear “pattern 2.” To perform a simulated psychophysical experiment, STRF distances could have been computed on a series of repeated trials using a distracter presented starting with a random modulating phase and a d' statistic could have been calculated from the distribution of the trial-by-trial distances. Such simulation, unfortunately, would have required computational resources that were not available. As a substitute, distances were computed between STRF patterns generated by the two targets embedded into a distracter having fixed magnitude and phase spectra. The targets were the single-formant FM patterns used in the experiments and illustrated in Figure 1; three of the five FM excursions used in the experiments were used in the simulation (10, 20, and 55% formant peak change with respect to the 1-kHz resting formant peak); the f0 of the target was always higher than that of the distracter and a single fundamental frequency difference Δf0 of 0.77 (the larger of the two tested in the experiments) was used. Figure 4 illustrates STRF time-frequency response patterns to the 55% targets in the distracter presented at 0 dB SNR. The two STRF's at the left show the upward and downward grating responses to the up-down pattern, whereas the rightmost panel shows time-frequency distances between the up-down and the down-up targets. Because the two stimuli differed only in their middle 200 ms portions (see Figure 1), the time range in the pictures contains 400 ms starting 100 ms before and finishing 100 ms after the FM portion of the targets.

**Figure 5:**
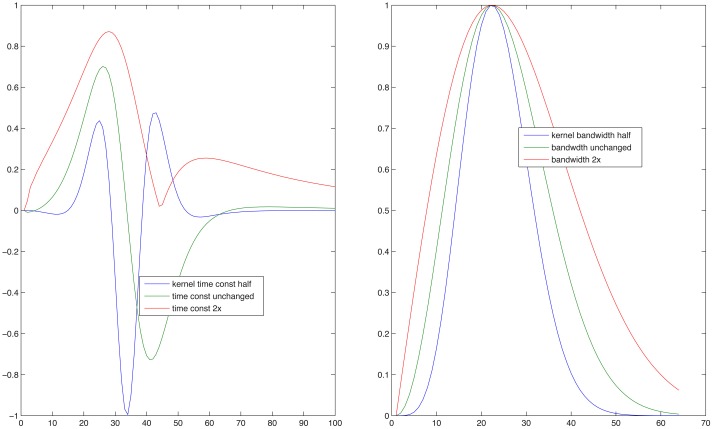
**Kernel functions used by the model of Chi et al. (2005) to generate temporal (left) and spectral (right) modulation planes at any given temporal rate and spectral scale specified**. The line in the center of each of the three shown in the two panels is the unmodified kernel function and was used to simulate results of the young subjects. In both panels the lines showing functions narrower than the central one were modified to produce temporal or spectral resolutions higher than those that supposedly underlay the good performance by the young listeners, whereas the lines outlining a broader function were modified to produce lower temporal or spectral resolutions that were expected to simulate results of the elderly subjects.

**Figure 6:**
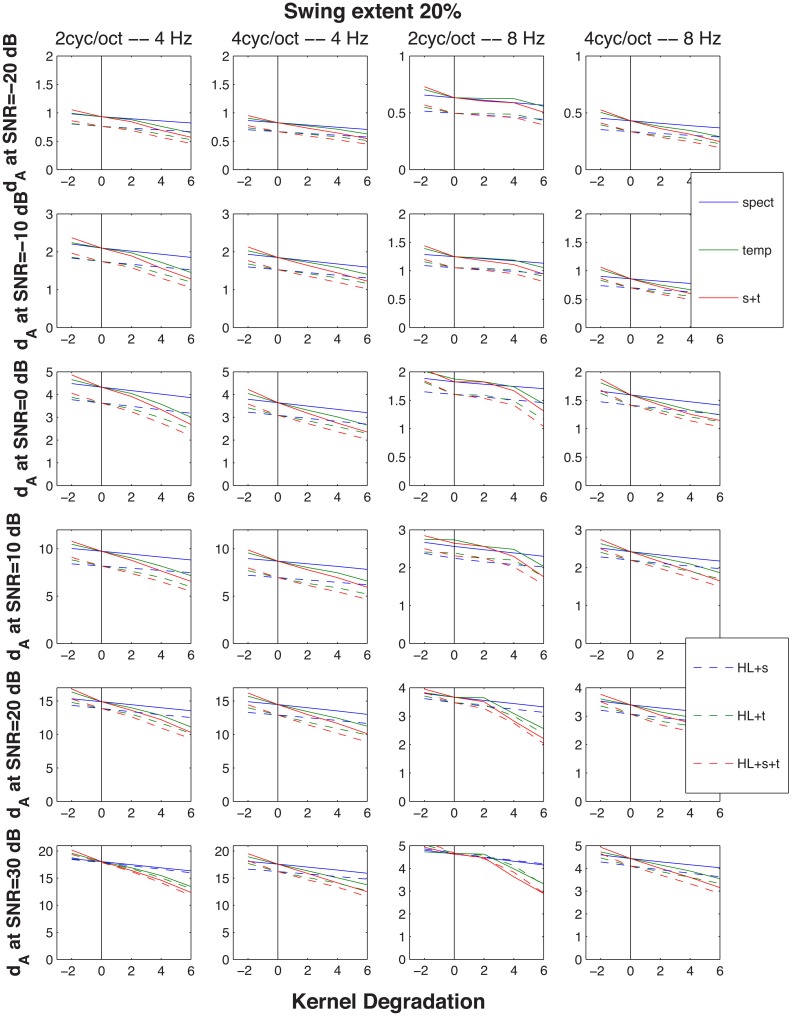
**Cumulative normalized Euclidean distances d_A_ between STRF patterns generated by the up-down and the down-up targets presented in an identical (magnitude and phase) distracter and identical stimulus parameters (extent of FM swing and SNR)**. The distance metric is considered to be proportional to the magnitude of the perceptual difference between the two targets, hence its increasing magnitude with SNR indicated in the six rows of graphs. The abscissa indicates the size of degradation (i.e., the size of change of the kernel function): 6 dB corresponds to a 2-fold increase in the function's broadness and the vertical line at 0 marks the point at which the original, un-modified kernel functions were used, resulting in identification performance as good as demonstrated by the young subjects. The lines with negative slope show simulation by modification of the functions affecting resolution of the spectral, the temporal, or both the spectral and temporal modulations. Solid lines were generated with only the STRF kernels modified, whereas the broken lines resulted from entering the STRF module with a stimulus low-pass filtered and with a slightly reduced frequency selectivity, both intended to reflect changes encountered in elderly individuals with a moderate presbycusic sensorineural hearing loss. The leftmost tail of the lines indicate a point generated by an improved, rather than degraded, kernel function—hence the higher distance (e.g., better identification performance) it is associated with. The four columns represent the four combinations of two temporal modulation rates (4 and 8 Hz) and two spectral modulation scales (2 and 4 cycles/octave). Across all conditions in the figure a single extent of FM excursion, 20% maximum counting from the 1 kHz resting formant frequency, was used.

**Figure 7:**
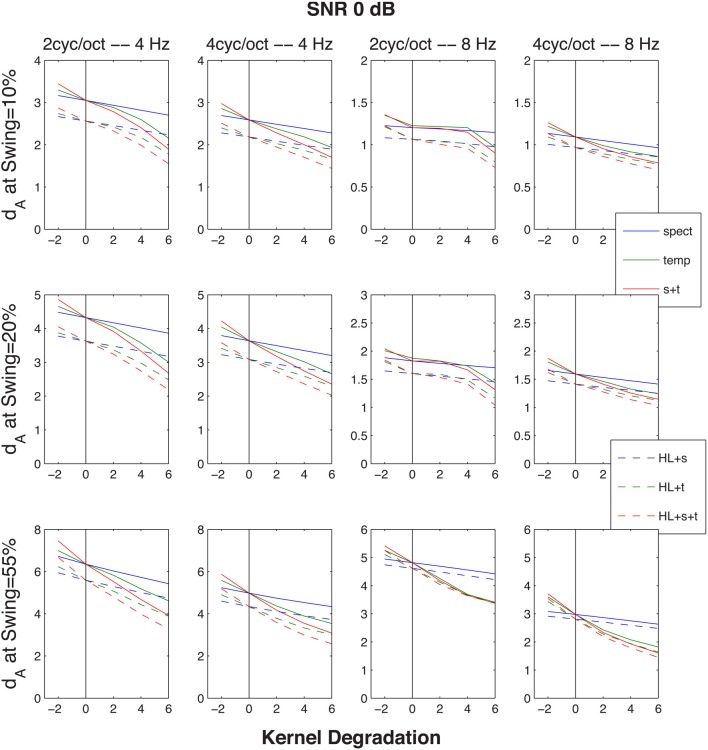
**Similar to Figure 6, except that the cumulative normalized Euclidean distances d_A_ between STRF patterns generated by the up-down and the down-up targets were evoked by the same target-distracter SNR of 0 dB, across three FM excursion extents (10, 20, and 55% maximum deflection re/the 1-kHz resting formant frequency), one for each row of graphs**. The abscissa and the ordinate are the same as in Figure 6 (distance vs. size of kernel degradation). The four columns of graphs represent the same four rate-cycle combinations (4, 8 Hz and 2, 4 cycles) as in Figure 6.

Since the objective of this simulation was to compare the model's predictions for normal and impaired listeners, auditory processing by elderly individuals was modeled in two ways. First, the typical high-frequency sloping hearing loss was emulated by passing the stimulus through a low-pass filter with a 1600-Hz corner frequency and a 6 dB/octave slope. In addition, the auditory spectrogram's filtering was made 10 percent less sharp, in order to mimic the broadening of cochlear frequency response often associated with age (Sommers and Gehr, 1998). Second, the spread of temporal (Takahashi and Bacon, 1992) and spectral (Sabin et al., 2013) modulation filters in aging was emulated by broadening the modulation filter kernels [the analogs to the Gabor (1946) transform's kernel] in the STRF model. This broadening of the two kernels is illustrated in Figure 5. For the data simulation, three different degrees of broadening (corresponding to factors of 1.26, 1.56, and 2, i.e., 2, 4, and 6 dB) and one degree of sharpening (a factor of 0.8, i.e., −2 dB) was used, either for the spectral, for the temporal, or both the spectral and temporal modulation filters. Results of these operations are illustrated in Figure 6 showing d_A_, the normalized (using standard deviations) cumulative Euclidean distance measure. These distances were computed between corresponding pixels of the time-frequency plane across SNR's ranging from −20 to 30 dB at one selected FM swing (20%) and are shown as a function of the degree of modulation filter degradation (with one negative degradation, i.e., improvement, as the leftmost point on each graph). The four columns of the figure display the distances for the four combinations of two the degrees of temporal modulation (2 and 4 Hz) and two degrees pf spectral modulation (2 and 4 cycles/octave). These modulation degrees were seen as being the most sensitive to the stimuli used in the study. Each graph illustrates the effect of two sets of simulations, one (solid lines) for an intact first stage (=auditory spectrogram) input to the STRF stage, and one (broken lines) for the case in which the first stage contained a presbycusic analog low-pass filter. The difference between no-hearing-loss solid lines and the presbycusic-loss broken lines gauges the effect of the simulated peripheral hearing loss, best seen where they cross the vertical line indicating the condition in which the modulation filters were left undegraded. Such peripheral loss effect is present across all SNR's and all four rate/cycle combinations, although its size varies (between about 5% to more than 25%) and it is generally smaller for large SNR's. Comparing the three types of modulation filter degradation, combined widening of the spectral and temporal filters was the most destructive, widening of the spectral filter alone had the least effect, and widening of the temporal filter alone took place in the middle. The largest degradation effect, over 50%, is associated with a 6 dB (i.e., doubled) increase of the parameter controlling spectral and temporal modulation filter width was seen for high-SNR low-pass filtered (i.e., presbycusic) stimuli. Similar observations can be made when looking at Figure 7 in which distance metrics across the three FM excursion extents are shown at the 0 dB SNR condition. Due to the relatively quiet signal level, absolute distance magnitudes and degradation effects are smaller than those seen in Figure 6.

**Figure 8:**
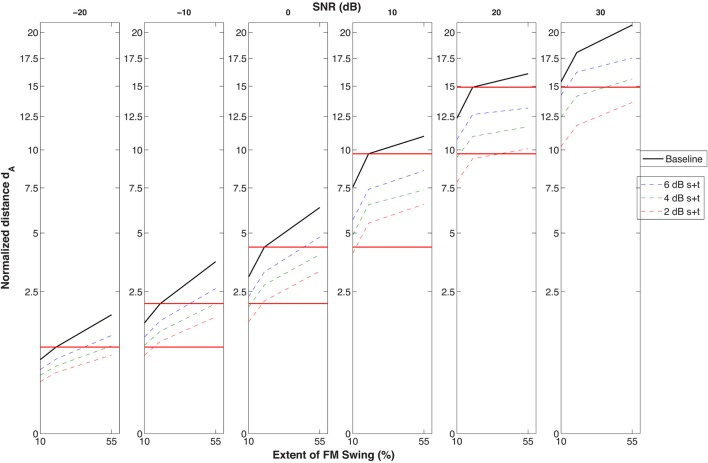
**Data simulating the almost-full set of conditions used in the identification experiments: identifiability of the targets (i.e., the cumulative normalized Euclidean distance between the STRF's evoked by the targets in distracter) as a function of three FM swing extents (10, 20, and 55%) across six SNR's**. The figure wishes to illustrate that, according to this simulation at least, the distance obtained by the un-degraded STRF (simulating the young subject) at he 20% swing extent in the −20 to +20 dB SNR conditions is approximately equivalent to the distance obtained at a SNR 10 dB higher by the simulated elderly having a moderate presbycusic hearing loss and STRF's generated by spectral and temporal kernels twice their normal size, i.e., kernels that produced a markedly reduced resolution of both spectral and temporal modulations. This 10 dB loss, marked by the horizontal red lines, goes in the direction indicated by the experimental results shown in Figure 2, although it does not reach the 20 dB difference obtained in the psychophysical experiments.

Although the methods used and the d_A_ metric adopted were not optimally suited for simulating psychometric functions, a graphic projection of the effect of degradation on SNR was attempted in Figure 8. In this figure the d_A_ scale was used to allow comparison of data across SNR's and across the three FM excursion conditions (10, 20, and 55%). The performance of presbycusic-filtered inputs to the second stage that underwent the three types of degradation (broken lines) is compared with the no-filtering-no-degradation condition taken as the baseline (dark black line, emulating our young subjects). The 20% excursion taken as the criterion projected to the next, easier 10 dB higher SNR condition shows that although that particular performance level is exceeded by the baseline, it is within the range of degraded STRF processors. For instance, we see that a −10 dB SNR for the most degraded (thin red line) condition with an (interpolated) excursion between 10 and 20% will lead to a performance level identical to that of a 10 dB less loud stimulus at a 20% FM swing going through an intact (unfiltered/un-degraded) model. Similar 10 dB (or near 10 dB) effects comparing the baseline with the most degraded condition can be seen at all SNR's. While this difference between undegraded-normal and degraded-impaired simulated subjects is smaller than the 20 dB drop in performance by the elderly compared to the young in the experiments, it still suggests that a degradation *of the cortical processor* responsible for modulation filtering may at least partially account for the age effect seen.

Aside the age effect the objective was to simulate, there are some valuable hints offered by the STRF model data. As expected, one can see in both Figures 6, 7 that those stimuli that easier to discriminate (such as larger SNR's and larger FM swings) produce larger or much larger distances. There is, however, a potentially more important observation. Although the stimuli were not speech, they were tailored with parameters reflecting the spectral and temporal dynamics of speech. Thus, it may not be without relevance to speech processing in the cortex that the largest distances were seen for the 4-Hz temporal modulation filter at both the 2- and the 4-cycles/octave spectral modulation filter. This indicates that the most active temporal modulation filter coincides with the most prominent 4-Hz modulation rate observed for conversational speech across talkers and across languages (Greenberg et al., 2003) and that the 2-to-4-cycles/octave spectral grating coincides with distances between peaks in the spectrum that are optimal for resolving formants and formant changes in vowels (Kewley-Port and Zheng, 1999).

Despite the fact that the simulation discussed in this subsection provides only an imperfect analogy to a psychophysical experiment, it has been able to provide an answer to the question that led us to emulating the data presented in the previous sections with the help of a well-established model—the STRF—that includes peripheral and central stages of auditory processing. This simulation appears to suggest that the deficit shown by the elderly listeners in the experiments is due to two factors. The first is a peripheral loss affecting frequency and temporal resolution, but only to a lesser degree than the second. This second, more potent factor is a deficiency in the resolution of temporal and spectral modulations performed by the auditory system at a more central site, most likely in the cortex. To better understand the role of this brain mechanism in the perception of everyday speech, work should be directed toward extracting principal *features* of STRF activity and the *trajectory* of those features over time. Such future work would allow us to get a better grip on mechanisms likely to be responsible for the CPE and its decay with age.

### Conflict of interest statement

The author declares that the research was conducted in the absence of any commercial or financial relationships that could be construed as a potential conflict of interest.

## Supplementary material

**Audio examples—to be listened to while looking at Figure 1**.

Audio example 1 Ascending–descending target formant FM pattern (left pattern of top trace in Figure 1). High fundamental frequency (189 Hz) with an excursion wider than the widest used in the experiment (75%). The FM rate is 5 Hz.

Audio example 2 Similar, but for the descending–ascending target formant FM pattern (right pattern of top trace in Figure 1).

Audio example 3 5-Hz FM pattern of the distracter. Low fundamental frequency (107 Hz). The formant swing is between the 525 and 100 Hz peak frequencies.

Audio example 4 A waveform similar to whose presented to the listener in the identification experiment (Experiment 1). It is, actually, the pattern of Audio example 2 presented in the middle of the distracter. The SPLs of the target and of the distracter in this example are identical. This large target-to-distracter ratio, together with the target's formant swing larger than those used in the experiment, produce a stimulus in which the target pattern would have been easily identified by all listeners in the study.

## References

[B1] Abel S. M. Giguere C. Consoli A. Papsin B. C. (2000). The effect of aging on horizontal plane sound localization. J. Acoust. Soc. Am. 108, 743–752 10.1121/1.429607 10955641

[B2] Agus T. R. Akeroyd M. A. Gatehouse S. Warden D. (2009). Informational masking in young and elderly listeners for speech masked by simultaneous speech and noise. J. Acoust. Soc. Am. 126, 1926–1940 10.1121/1.3205403 19813805

[B3] Arai T. Greenberg S. (1997). The temporal properties of spoken japanese are similar to those of English, in Paper presented at the Eurospeech (Rhodes).

[B4] Arbogast T. L. Mason C. R. Kidd J. G. (2002). The effect of spatial separation on informational and energetic masking of speech. J. Acoust. Soc. Am. 112, 2086–2098 10.1121/1.1510141 12430820

[B5] Assmann P. F. Summerfield A. Q. (1990). Modeling the perception of concurrent vowels: vowels with different fundamental frequencies. J. Acoust. Soc. Am. 88, 680–697 10.1121/1.399772 2212292

[B6] Bacon S. P. Takahashi G. A. (1992). Overshoot in normal-hearing and hearing-impaired subjects. J. Acoust. Soc. Am. 91, 2865–2871 10.1121/1.402967 1629479

[B7] Bregman A. S. (1990). Auditory Scene Analysis: The Perceptual Organization of Sound. Cambridge, MA: Bradford Books (MIT Press)

[B8] Brungart D. S. Simpson B. D. (2002). Within-ear and across-ear interference in a cocktail-party listening task. J. Acoust. Soc. Am. 112, 2985–2995 10.1121/1.1512703 12509020

[B9] Brungart D. S. Simpson B. D. (2007). Effect of target-masker similarity on across-ear interference in a dichotic cocktail-party listening task. J. Acoust. Soc. Am. 122, 1724 10.1121/1.2756797 17927432

[B10] Brungart D. S. Simpson B. D. Ericson M. A. Scott K. R. (2001). Informational and energetic masking effects in the perception of multiple simultaneous talkers. J. Acoust. Soc. Am. 110, 2527–2538 10.1121/1.1408946 11757942

[B11] Carhart R. Tillman T. W. (1970). Interaction of competing speech signals with hearing losses. Arch. Otolaryngol. 91, 273–279 10.1001/archotol.1970.00770040379010 5414080

[B12] Chi T. Ru P. Shamma S. A. (2005). Multiresolution spectrotemporal analysis of complex sounds. J. Acoust. Soc. Am. 118, 887–906 10.1121/1.1945807 16158645

[B13] Cooke M. Garcia Lecumberri M. L. Barker J. (2008). The foreign language cocktail party problem: energetic and informational masking effects in non-native speech perception. J. Acoust. Soc. Am. 123, 414–427 10.1121/1.2804952 18177170

[B14] Darwin C. J. Brungart D. S. Simpson B. D. (2003). Effects of fundamental frequency and vocal-tract length changes on attention to one of two simultaneous talkers. J. Acoust. Soc. Am. 114, 2913–2922 10.1121/1.1616924 14650025

[B15] Darwin C. J. Hukin R. W. (1998). Perceptual segregation of a harmonic from a vowel by interaural time difference in conjunction with mistuning and onset asynchrony. J. Acoust. Soc. Am. 103, 1080–1084 10.1121/1.421221 9479762

[B16] Divenyi P. (2005). Masking the feature-information in multi-stream speech-analogue displays, in Speech Separation by Humans and Machines, ed Divenyi P. (New York, NY: Kluwer Academic Publishers), 269–281

[B17] Divenyi P. (2009). Perception of complete and incomplete formant transitions in vowels. J. Acoust. Soc. Am. 126, 1427–1439 10.1121/1.3167482 19739756 PMC2809691

[B19] Divenyi P. L. Haupt K. M. (1997). Audiological correlates of speech understanding deficits in elderly listeners with mild-to-moderate hearing loss. I. Age and laterality effects. Ear Hear. 18, 42–61 10.1097/00003446-199702000-00005 9058037

[B20] Dubno J. R. Dirks D. D. Morgan D. E. (1984). Effects of age and mild hearing loss on speech recognition in noise. J. Acoust. Soc. Am. 76, 87–96 10.1121/1.391011 6747116

[B21] Durlach N. I. Mason C. R. Kidd G. Jr. Arbogast T. L. Colburn H. S. Shinn-Cunningham B. G. (2003a). Note on informational masking. J. Acoust. Soc. Am. 113, 2984–2987 10.1121/1.1570435 12822768

[B22] Durlach N. I. Mason C. R. Shinn-Cunningham B. G. Arbogast T. L. Colburn H. S. Kidd G. Jr. (2003b). Informational masking: counteracting the effects of stimulus uncertainty by decreasing target-masker similarity. J. Acoust. Soc. Am. 114, 368–379 10.1121/1.1577562 12880048

[B23] Elhilali M. Shamma S. A. (2008). A cocktail party with a cortical twist: how cortical mechanisms contribute to sound segregation. J. Acoust. Soc. Am. 124, 3751–3771 10.1121/1.3001672 19206802 PMC2676630

[B24] Elliott L. L. Hammer M. A. Scholl M. E. Wasowicz J. M. (1989). Age differences in discrimination of simulated single-formant frequency transitions. Percept. Psychophys. 46, 181–186 10.3758/BF03204981 2762106

[B25] Elliott T. M. Theunissen F. E. (2009). The modulation transfer function for speech intelligibility. PLoS Comput. Biol. 5:e1000302 10.1371/journal.pcbi.1000302 19266016 PMC2639724

[B26] Ezzatian P. Li L. Pichora-Fuller K. Schneider B. (2011). The effect of priming on release from informational masking is equivalent for younger and older adults. Ear Hear. 32, 84–96 10.1097/AUD.0b013e3181ee6b8a 21178568

[B27] Fitzgibbons P. J. Gordon-Salant S. (1994). Age effects on measures of auditory duration discrimination. J. Speech Hear. Res. 37, 662–670 8084196 10.1044/jshr.3703.662

[B28] Fitzgibbons P. J. Gordon-Salant S. (1998). Auditory temporal order perception in younger and older adults. J. Speech Lang. Hear. Res. 41, 1052–1060 9771628 10.1044/jslhr.4105.1052

[B29] Fogerty D. Kewley-Port D. (2009). Perceptual contributions of the consonant-vowel boundary to sentence intelligibilitya). J. Acoust. Soc. Am. 126, 847–857 10.1121/1.3159302 19640049 PMC2730717

[B30] Freyman R. L. Balakrishnan U. Helfer K. S. (2004). Effect of number of masking talkers and auditory priming on informational masking in speech recognition. J. Acoust. Soc. Am. 115, 2246–2256 10.1121/1.1689343 15139635

[B31] Freyman R. L. Balakrishnan U. Helfer K. S. (2008). Spatial release from masking with noise-vocoded speech. J. Acoust. Soc. Am. 124, 1627–1637 10.1121/1.2951964 19045654 PMC2736712

[B32] Gabor D. (1946). Theory of communication. J. Inst. Elec. Eng. 93, 429–457 23890580

[B33] Gelfand S. A. Ross L. Miller S. (1988). Sentence reception in noise from one versus two sources: effect of aging and hearing loss. J. Acoust. Soc. Am. 83, 248–256 10.1121/1.396426 3343444

[B34] Gordon-Salant S. Fitzgibbons P. J. (1999). Profile of auditory temporal processing in older listeners. J. Speech Lang. Hear. Res. 42, 300–311 10229448 10.1044/jslhr.4202.300

[B35] Gordon-Salant S. Fitzgibbons P. J. Friedman S. A. (2007). Recognition of time-compressed and natural speech with selective temporal enhancements by young and elderly listeners. J. Speech Lang. Hear. Res. 50, 1181–1193 10.1044/1092-4388(2007/082) 17905904

[B36] Greenberg S. Carvey H. M. Hitchcock L. Chang S. (2003). Temporal properties of spontaneous speech – A syllable-centric perspective. J. Phonetics 31, 465–485 10.1016/j.wocn.2003.09.005

[B37] Greenwood D. D. (1962). Approximate calculation of the dimensions of travelingwave envelopes in four species. J. Acoust. Soc. Am. 34, 1364–1369 10.1121/1.1918349

[B38] Hawley M. L. Litovsky R. Y. Culling J. F. (2004). The benefit of binaural hearing in a cocktail party: effect of location and type of interferer. J. Acoust. Soc. Am. 115, 833–843 10.1121/1.1639908 15000195

[B39] He N. J. Mills J. H. Ahlstrom J. B. Dubno J. R. (2008). Age-related differences in the temporal modulation transfer function with pure-tone carriers. J. Acoust. Soc. Am. 124, 3841–3849 10.1121/1.2998779 19206810 PMC2676625

[B40] Herman G. E. Warren L. R. Wagener J. W. (1977). Auditory lateralization: age differences in sensitivity to dichotic time and amplitude cues. J. Gerontol. 32, 187–191 10.1093/geronj/32.2.187

[B41] Hillenbrand J. M. Clark M. J. Nearey T. M. (2001). Effects of consonant environment on vowel formant patterns. J. Acoust. Soc. Am. 109, 748–763 10.1121/1.1337959 11248979

[B42] Humes L. E. Dubno J. R. Gordon-Salant S. Lister J. J. Cacace A. T. Cruickshanks K. J. (2012). Central presbycusis: a review and evaluation of the evidence. J. Am. Acad. Audiol. 23, 635–666 10.3766/jaaa.23.8.5 22967738 PMC5898229

[B43] Humes L. E. Kidd G. R. Lentz J. J. (2013). Auditory and cognitive factors underlying individual differences in aided speech-understanding among older adults. Front. Syst. Neurosci. 7:55 10.3389/fnsys.2013.00055 24098273 PMC3787592

[B44] Humes L. E. Watson B. U. Christensen L. A. Cokely C. G. Halling D. C. Lee L. (1994). Factors associated with individual differences in clinical measures of speech recognition among the elderly. J. Speech Hear. Res. 37, 465–474 8028328 10.1044/jshr.3702.465

[B45] Ihlefeld A. Shinn-Cunningham B. (2008). Spatial release from energetic and informational masking in a divided speech identification task. J. Acoust. Soc. Am. 123, 4380–4392 10.1121/1.2904825 18537389 PMC9014250

[B46] Kewley-Port D. Zheng Y. (1999). Vowel formant discrimination: towards more ordinary listening conditions. J. Acoust. Soc. Am. 106, 2945–2958 10.1121/1.428134 10573907

[B47] Kidd G. Jr. Mason C. R. Deliwala P. S. Woods W. S. Colburn H. S. (1994). Reducing informational masking by sound segregation. J. Acoust. Soc. Am. 95, 3475–3480 10.1121/1.410023 8046139

[B48] Kowalski N. Depireux D. A. Shamma S. A. (1996). Analysis of dynamic spectra in ferret primary auditory cortex. I. Characteristics of single-unit responses to moving ripple spectra. J. Neurophysiol. 76, 3503–3523 8930289 10.1152/jn.1996.76.5.3503

[B49] Lavandier M. Culling J. F. (2008). Speech segregation in rooms: monaural, binaural, and interacting effects of reverberation on target and interferer. J. Acoust. Soc. Am. 123, 2237–2248 10.1121/1.2871943 18397029

[B50] Lee J. H. Humes L. E. (2012). Effect of fundamental-frequency and sentence-onset differences on speech-identification performance of young and older adults in a competing-talker background. J. Acoust. Soc. Am. 132, 1700–1717 10.1121/1.4740482 22978898 PMC3460987

[B51] Lee T. Y. Richards V. M. (2011). Evaluation of similarity effects in informational masking. J. Acoust. Soc. Am. 129, EL280–EL285 10.1121/1.3590168 21682365 PMC3117891

[B52] Levi A. Stark H. (1983). Signal restoration from phase by projection onto a convex set. J. Opt. Soc. Am. 73, 810–822 10.1364/JOSA.73.000810

[B53] Lutfi R. A. (1990). How much masking is informational masking? J. Acoust. Soc. Am. 88, 2607–2610 10.1121/1.399980 2283433

[B54] Lyzenga J. Carlyon R. P. (1999). Center frequency modulation detection for harmonic complexes resembling vowel formants and its interference by off-frequency maskers. J. Acoust. Soc. Am. 105, 2792–2806 10.1121/1.426896 10335631

[B55] Lyzenga J. Carlyon R. P. (2005). Detection, direction discrimination, and off-frequency interference of center-frequency modulations and glides for vowel formants. J. Acoust. Soc. Am. 117, 3042–3053 10.1121/1.1882943 15957773

[B56] McAdams S. (1990). Interactions among cues contributing to concurrent sound segregation. J. Acoust. Soc. Am. 88, S146 10.1016/j.heares.2009.09.012

[B57] Mesgarani N. David S. V. Fritz J. B. Shamma S. A. (2008). Phoneme representation and classification in primary auditory cortex. J. Acoust. Soc. Am. 123, 899–909 10.1121/1.2816572 18247893

[B58] Mesgarani N. Thomas S. Hermansky H. (2011). Toward optimizing stream fusion in multistream recognition of speech. J. Acoust. Soc. Am. 130, EL14–EL18 10.1121/1.3595744 21786862

[B59] Micheyl C. Oxenham A. J. (2010). Pitch, harmonicity and concurrent sound segregation: psychoacoustical and neurophysiological findings. Hear. Res. 266, 36–51 10.1016/j.heares.2009.09.012 19788920 PMC2885481

[B60] Moore B. C. Sek A. Shailer M. J. (1995). Modulation discrimination interference for narrow-band noise modulators. J. Acoust. Soc. Am. 97, 2493–2497 10.1121/1.411969 7714266

[B61] Moore B. C. J. Sek A. (1996). Detection of frequency modulation at low modulation rates: evidence for a mechanism based on phase locking. J. Acoust. Soc. Am. 100, 2320–2331 10.1121/1.417941 8865639

[B62] Murphy D. R. Craik F. I. Li K. Z. Schneider B. A. (2000). Comparing the effects of aging and background noise on short-term memory performance. Psychol. Aging 15, 323–334 10.1037/0882-7974.15.2.323 10879586

[B63] Ng E. H. Rudner M. Lunner T. Pedersen M. S. Ronnberg J. (2013). Effects of noise and working memory capacity on memory processing of speech for hearing-aid users. Int. J. Audiol. 52, 433–441 10.3109/14992027.2013.776181 23550584

[B64] Oh E. L. Lutfi R. A. (1998). Nonmonotonicity of informational masking. J. Acoust. Soc. Am. 104, 3489–3499 10.1121/1.423932 9857508

[B65] Patterson R. Moore B. (1986). Auditory filters and excitation patterns as representations of frequency resolution, in Frequency Selectivity in Hearing, ed Moore B. (London: Academic Press), 123–178

[B66] Pitton J. W. Wang K. Juang B.-H. (1996). Time-frequency analysis and auditory modeling for automatic recognition of speech. Proc. IEEE 84, 1199–1215 10.1109/5.535241

[B67] Plomp R. (1983). Perception of speech as a modulated signal, in Proceedings of the 10th International Congress of Phonetic Science, M. P. R. van der Broecke and A. Cohen (Dordrecht: Foris), 29–40

[B68] Rajan R. Cainer K. E. (2008). Ageing without hearing loss or cognitive impairment causes a decrease in speech intelligibility only in informational maskers. Neuroscience. 154, 784–795 10.1016/j.neuroscience.2008.03.067 18485606

[B69] Roman N. Wang D. (2006). Pitch-based monaural segregation of reverberant speech. J. Acoust. Soc. Am. 120, 458–469 10.1121/1.2204590 16875242

[B70] Sabin A. T. Clark C. A. Eddins D. A. Wright B. A. (2013). Different patterns of perceptual learning on spectral modulation detection between older hearing-impaired and younger normal-hearing adults. J. Assoc. Res. Otolaryngol. 14, 283–294 10.1007/s10162-012-0363-y 23229719 PMC3660913

[B71] Shackleton T. M. Carlyon R. P. (1994). The role of resolved and unresolved harmonics in pitch perception and frequency modulation discrimination. J. Acoust. Soc. Am. 95, 3529–3540 10.1121/1.409970 8046144

[B72] Singh N. C. Theunissen F. E. (2003). Modulation spectra of natural sounds and ethological theories of auditory processing. J. Acoust. Soc. Am. 114, 3394–3411 10.1121/1.1624067 14714819

[B73] Snell K. B. Frisina D. R. (2000). Relationships among age-related differences in gap detection and word recognition. J. Acoust. Soc. Am. 107, 1615–1626 10.1121/1.428446 10738815

[B74] Snell K. B. Mapes F. M. Hickman E. D. Frisina D. R. (2002). Word recognition in competing babble and the effects of age, temporal processing, and absolute sensitivity. J. Acoust. Soc. Am. 112, 720–727 10.1121/1.1487841 12186051

[B75] Sommers M. S. (1997). Stimulus variability and spoken word recognition. II. The effects of age and hearing impairment. J. Acoust. Soc. Am. 101, 2278–2288 10.1121/1.418208 9104029

[B76] Sommers M. S. Gehr S. E. (1998). Auditory suppression and frequency selectivity in older and younger adults. J. Acoust. Soc. Am. 103, 1067–1074 10.1121/1.421220 9479760

[B77] Takahashi G. A. Bacon S. P. (1992). Modulation detection, modulation masking, and speech understanding in noise in the elderly. J. Speech Hear. Res. 35, 1413–1429 1494284 10.1044/jshr.3506.1410

[B78] Terhardt E. Stoll G. Seewann M. (1982). Algorithm for extraction of pitch and pitch salience from complex tonal signals. J. Acoust. Soc. Am. 71, 679–688 10.1121/1.387544 6699300

[B79] Watson C. S. (1987). Uncertainty, informational masking, and the capacity of immediate auditory memory, in Auditory Processing of Complex Sounds, ed Yost W. A. Watson C. S. (Hillsdale, NJ: L. Erlbaum), 267–287

[B80] Zekveld A. A. Kramer S. E. Festen J. M. (2011). Cognitive load during speech perception in noise: the influence of age, hearing loss, and cognition on the pupil response. Ear Hear. 32, 498–510 10.1097/AUD.0b013e31820512bb 21233711

